# Supplementary material for: Effect of Cooking and in vitro Digestion on the Polyphenols and Antioxidant Properties of Asparagus officinalis L. cultivars
Source: Foods. 2025 Jul 3;14(13):2367. doi: 10.3390/foods14132367 (PMC12249104; doi:10.3390/foods14132367)
Supplement: Supplementary file 1 [file foods-14-02367-s001.zip › foods-3724488-supplementary.pdf]

Table S1. Correlation between TPC and data obtained by the DPPH, ABTS and FRAP tests.

**Assay**	**R**			
	Placoseps		*Darlise*	
	**February 2024**	**April 2024**	**February 2024**	**April 2024**
**DPPH**	0.93	0.86	0.82	0.95
**ABTS**	0.93	0.73	1.00	1.00
**FRAP**	0.94	0.90	0.58	0.92

The correlation coefficients were evaluated by using Pearson's method.
